# Supplementary material for: Distantly related Alteromonas bacteriophages share tail fibers exhibiting properties of transient chaperone caps
Source: Nat Commun. 2023 Oct 16;14:6517. doi: 10.1038/s41467-023-42114-8 (PMC10579305; doi:10.1038/s41467-023-42114-8)
Supplement: Supplementary file 1 — Supplementary Information [file 41467_2023_42114_MOESM1_ESM.pdf]

Distantly related *Alteromonas* bacteriophages share tail fibers exhibiting properties of transient chaperone caps

Supplementary Information

Table S1. Confidence scores of AlphaFold-generated models.

Table S2. Oligonucleotides used to generate protein expression plasmids.

Table S3. Percent similarity in the nucleotide sequence alignments of the proteins located in the host recognition module of phages V22, A5 and P24.

Figure S1. Genome comparative analysis of several N4-like schitoviruses that share a giant RNA polymerase of ca. 10 kb in size.

Figure S2. AlphaFold-based analysis of additional phage components: the central fiber, gp41 “Dit” of P24, short tail fiber gp23 of V22, CheY-like gp11 of A5, and the *Salmonella* phage S16 tail fiber IMC.

Figure S3. Sequence and structure alignment of the knob and head domains of the indicated tail fibers.

Supplementary Tables

Phage	Protein	Program used	pLDDT (average)	Interface pTM	Figure
A5	gp8 (tail fiber)	Alphafold-Multimer	75.8	0.57	4
A5	gp9 (chaperone)	AlphaFold 2.0	94.8	n/a	4
A5	gp9 (chaperone)	Alphafold-Multimer	93.0	0.60	6
A5	gp8 Gly150 to Phe462 + gp9	Alphafold-Multimer	70.5	0.59	6*
V22	gp26 (tail fiber)	Alphafold-Multimer	77.4	0.67	4
V22	gp27 (chaperone)	AlphaFold 2.0	95.2	n/a	4
V22	gp27 (chaperone)	Alphafold-Multimer	84.8	0.21	6
V22	gp26 Gly160 to Phe369 + gp27	Alphafold-Multimer	87.8	0.89	6*
P24	gp44 (tail fiber)	Alphafold-Multimer	75.1	0.65	4
P24	gp45 (chaperone)	AlphaFold 2.0	94.5	n/a	4
GOV_bin2917	gp51 (tail fiber)	Alphafold-Multimer	65.5	0.46	4
GOV_bin2917	gp50 (chaperone)	AlphaFold 2.0	95.3	n/a	4
A5	gp6 (central fiber)	Alphafold-Multimer	81.5	0.54	S2
A5	gp11 (CheY-like)	AlphaFold 2.0	92.4	n/a	S2
V22	gp24 (central fiber)	Alphafold-Multimer	76.6	0.59	S2
V22	gp23 (short tail fiber)	Alphafold-Multimer	90.8	0.68	S2
P24	gp43 (central fiber)	Alphafold-Multimer	67.3	0.43	S2
P24	gp41 (Dit)	Alphafold-Multimer	76.1	0.51	S2
S16	gp37 Ala516 to Lys749 (tail fiber)	Alphafold-Multimer	94.3	0.92	S2

Table S1. Confidence scores of AlphaFold-generated models. Confidence per residue is calculated as a predicted Local Distance Difference Test score (0-100), with an average of all residues within the models provided below. A pLDDT ≥ 90 have very high model confidence, residues with 90 > pLDDT ≥ 70 are classified as confident, while residues with 70 > pLDDT > 50 have low confidence. Interface pTM scores ("iptm+ptm") are a measure of predicted structure accuracy generated by AlphaFold-Multimer and provide the overall confidence score for the complete model (scored 0 to 1). *Models kindly generated by Petr G. Leiman (The University of Texas Medical Branch at Galveston, USA).

Construct	Forward primer (5´- 3´)	Reverse primer (5´- 3´)
pQE30_HGT backbone	GCCCTGGAAATACAGATTCTCG	AATTAGCTGAGCTTGGACTCCT
gp8_gp9 insert	CGAGAATCTGTATTTCCAGGGCATGGCTAGTACATTTTGGATT	AGGAGTCCAAGCTCAGCTAATTTTACCAAGTTAAGTTTGCTAGATAT
gp8 insert	CGAGAATCTGTATTTCCAGGGCATGGCTAGTACATTTTGGATT	AGGAGTCCAAGCTCAGCTAATTTTAGAAGTTTACAGTGATTTTTGC

Table S2. Oligonucleotides used to generate protein expression plasmids.

		V22											A5				
		gp21	gp22	gp23	gp24	gp25	gp26	gp27	gp29	gp30	gp31	gp32	gp4	gp5	gp6	gp8	gp9
A5	gp4	---	n.s.s.	---	---	---	---	---	---	---	---	---	---	---	---	---	---
	gp5	---	---	n.s.s.	---	---	---	---	---	---	---	---	---	---	---	---	---
	gp6	---	---	---	69.05% Q.c. = 11%	---	---	---	---	---	---	---	---	---	---	---	---
	gp7	---	---	---	---	67.65% Q.c. = 82%	---	---	---	---	---	---	---	---	---	---	---
	gp8	---	---	---	---	---	58.70% Q.c. = 35%	---	---	---	---	---	---	---	---	---	---
	gp9	---	---	---	---	---	---	80.70% Q.c. = 100%	---	---	---	---	---	---	---	---	---
	gp10	---	---	---	---	---	---	---	50% Q.c. = 35%	---	---	---	---	---	---	---	---
	gp11	---	---	---	---	---	---	---	---	42.57% Q.c. = 86%	---	---	---	---	---	---	---
	gp12	---	---	---	---	---	---	---	---	---	57.14% Q.c. = 10%	---	---	---	---	---	---
	gp13	---	---	---	---	---	---	---	---	---	---	42.31% Q.c. = 89%	---	---	---	---	---
P24	gp40	n.s.s.	---	---	---	---	---	---	---	---	---	---	---	---	---	---	---
	gp41	---	n.s.s.	---	---	---	---	---	---	---	---	---	n.s.s.	---	---		---
	gp42	---	---	n.s.s.	---	---	---	---	---	---	---	---	---	n.s.s	---		---
	gp43	---	---	---	51.72% Q.c. = 2%	---	---	---	---	---	---	---	---	---	50.91% Q.c. = 68%	---	---
	gp44	---	---	---	---	---	61.54% Q.c. = 12%	---	---	---	---	---	---	---	---	42.86% Q.c. = 52%	---
	gp45	---	---	---	---	---	---	41.86% Q.c. = 21%	---	---	---	---	---	---	---	---	n.s.s.

Table S3. Percent similarity in the nucleotide sequence alignments of the proteins located in the host recognition module of phages V22, A5, and P24. Comparisons were done using the tBLASTx suite. Q.c.: Query cover.

Supplementary Figures

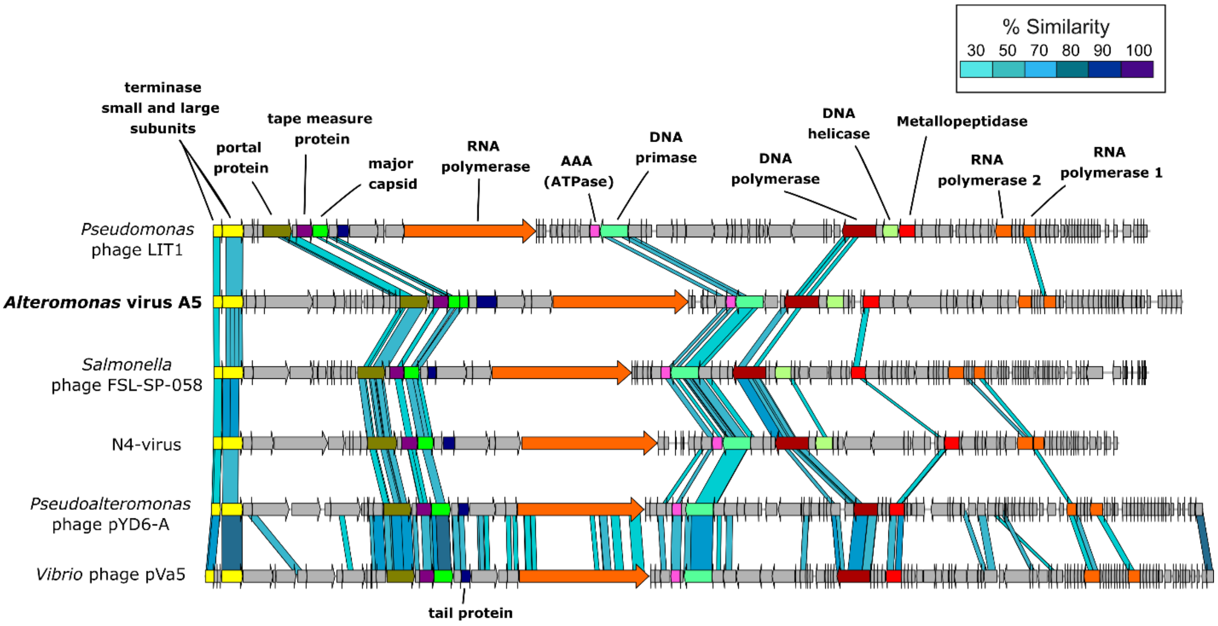

Figure S1. Genome comparative analysis of several N4-like schitoviruses that share a giant RNA polymerase of ca. 10 kb in size. Sequence comparisons were performed using tBLASTx with 30% minimal similarity on 100 bp minimum alignments. The host-recognition module of these phages did not show synteny or sequence similarity. Phage LIT1 (NC_013692), Phage A5 (OP481051), Phage FSL-SP-058 (NC_021772), Phage N4 (NC_008720), Phage Pyd6-a (NC_020849), Phage pVa5 (NC_049379).

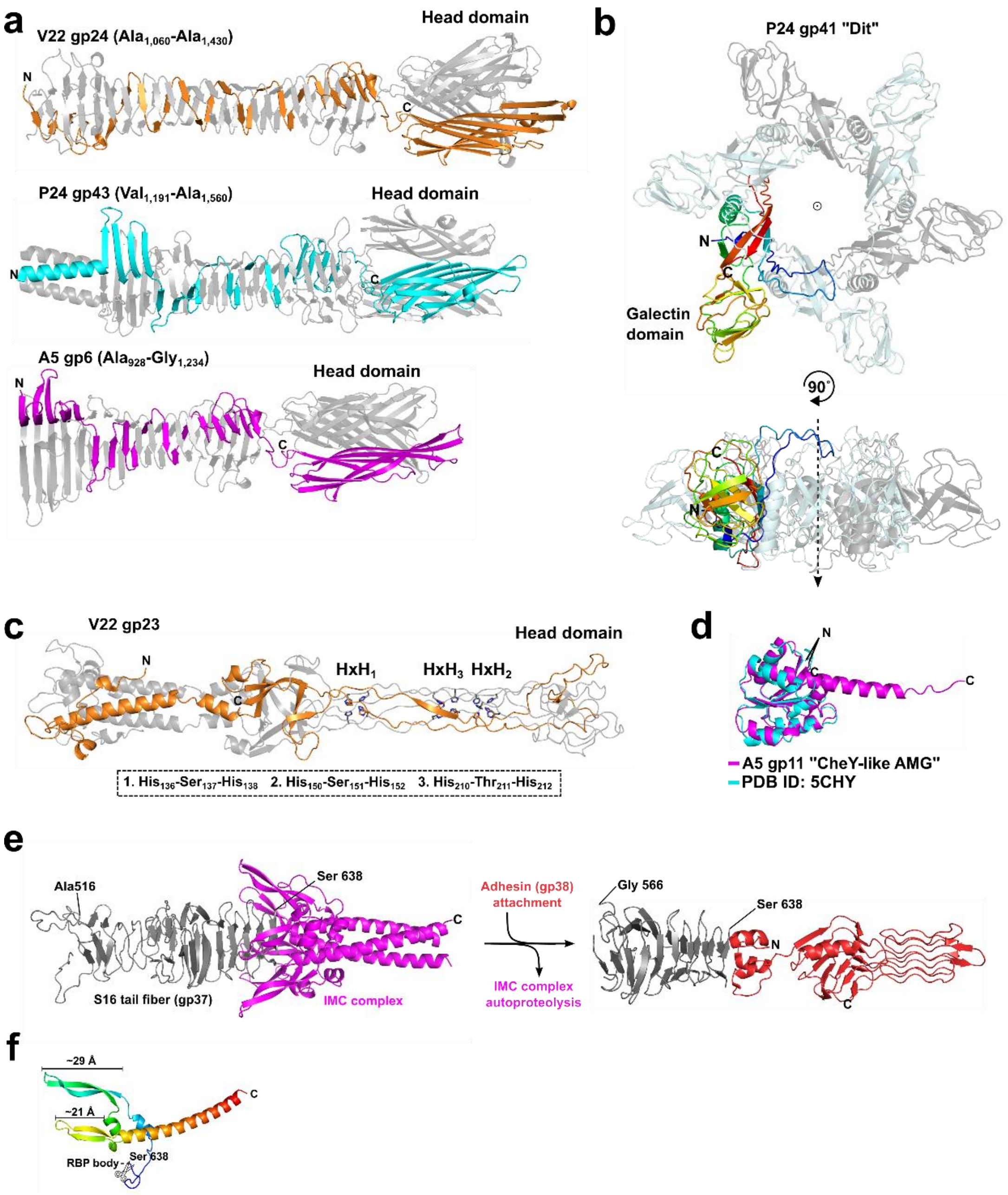

Figure S2. AlphaFold-based analysis of additional phage components: the central fiber, gp41 "Dit" of P24, short tail fiber gp23 of V22, CheY-like gp11 of A5, and the *Salmonella* phage S16 tail fiber IMC. **(A)** Ribbon diagrams of the homotrimeric C-terminal tips of the putative central RBP of phages V22 (gp24), P24 (gp43), and A5 (gp6) predicted using AlphaFold-Multimer. The distal head domains of all three central RBPs consists of extended lectin-like binding domains similar to the head domains of the phage's tail fibers (e.g., A5 gp8). **(B)** Ribbon diagram of the hexameric assembly of P24 gp41 predicted using AlphaFold-Multimer which resembles a classical "evolved" distal tail protein (Dit) complex, which is a highly conserved central building block of all Siphoviral baseplates. As highlighted, gp41 features a galectin-like carbohydrate-binding module (CBM) which is a characteristic feature of "evolved" Dit complexes. **(C)** Ribbon diagram of homotrimeric V22 gp23 as predicted using AlphaFold-Multimer features a classic phage T4 gp37-like tail fiber architecture (PDB ID: 2XGF) with an intertwined, elongated needle-like domain containing three HxH metal-binding sites that are expected to each be occupied by an Fe^{2+} ion. **(D)** Ribbon representations of A5 gp11 and *E.* coli chemotaxis protein CheY (PDB ID: 5CHY) superpose with a root-mean-square deviation (C-α RMSD) of 1.67 Å. **(E)** Ribbon diagram of the *Salmonella* phage S16 tail fiber (gp37) and its autoproteolytic C-terminal IMC predicted using AlphaFold-Multimer. The fiber tip associates with its adhesin (gp38) to form a mature, functional fiber (to the right, PDB ID: 6F45 [45]. **(F)** The gp37 IMC closely resembles the IMCs of the T5 and K1F RBPs (shown in **Fig. 6 A,C**), but was predicted as having an additional β-hairpin domain. Model confidence scores are provided in **Table S1**. Source Data files (.pdb) are provided.

a b

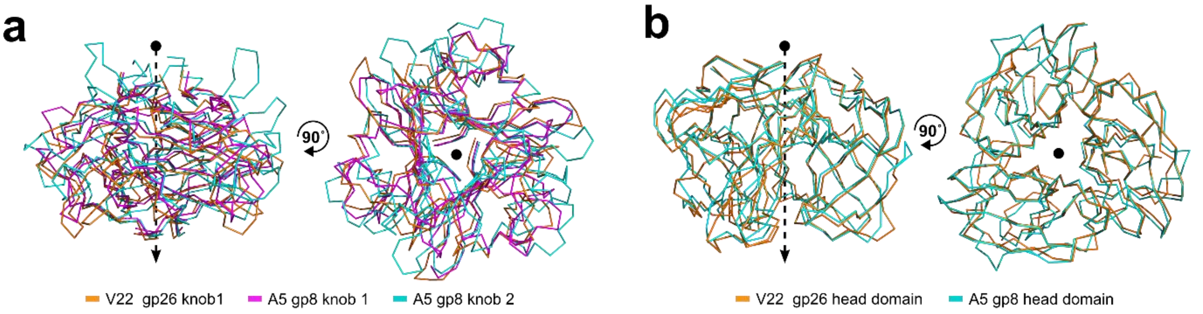

c

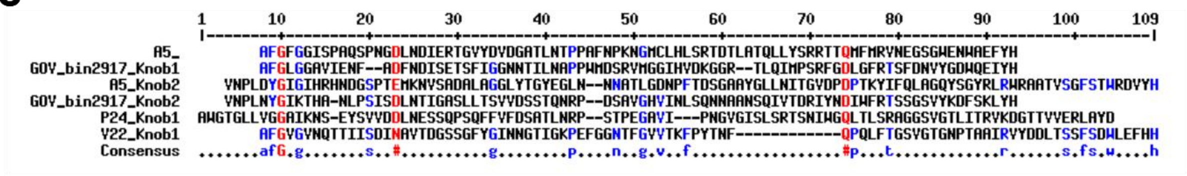

	V22_Knob1	A5_Knob2	GOV_Bin2917_Knob1	A5_Knob1	GOV_Bin2917_Knob2	P24_Knob1
V22_Knob1	100	17.24	20.51	19.51	18.99	17.72
A5_Knob2	17.24	100	21.52	16.87	18.39	14.29
GOV_Bin2917_Knob1	20.51	21.52	100	27.16	23.68	21.79
A5_Knob1	19.51	16.87	27.16	100	26.25	23.75
GOV_Bin2917_Knob2	18.99	18.39	23.68	26.25	100	25
P24_Knob1	17.72	14.29	21.79	23.75	25	100

Average Seq. Similarity = 20.8 % ± 2.8

d

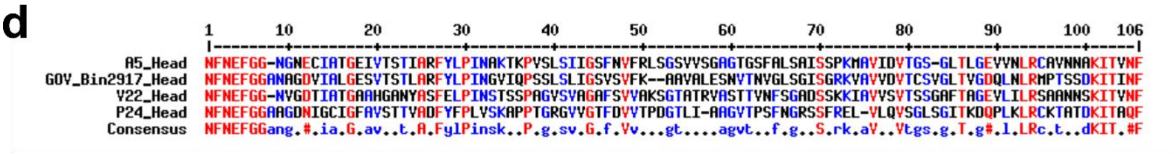

	P24_Head	GOV_Bin2917_Head	A5_Head	V22_Head
P24_Head	100	33.33	36.27	33.98
GOV_Bin2917_Head	33.33	100	55.88	41.75
A5_Head	36.27	55.88	100	49.04
V22_Head	33.98	41.75	49.04	100

Average Seq. Identity = 41.7 % ± 8.3

Figure S3. Sequence and structure alignment of the knob and head domains of the indicated tail fibers. (A) Superposition of the knob domain from V22 gp26 (orange) with the two knob domains of A5 gp8 (knob 1, magenta; knob 2, cyan) was performed using the DALI server [38]. Despite all knob domains sharing low (<20%) sequence identity, they feature the same domain structure with root-mean-square deviations (RMSD) of 1.5 Å and 2.3 Å for the V22 knob to knob 1 and knob 2 of A5, respectively. **(B)** Superposition of the distal head domains from V22 gp26 (orange) and A5 gp8 (cyan) also revealed they share the same domain structure with RMSD of 0.59 Å as was expected from 49% sequence similarity. MultAlin-generated [74] sequence alignments of all knob domains **(C)** and all distal head domains **(D)** from the four phage TFs described in this study with average sequence similarities determined using Clustal Omega [81]. All model confidence scores are provided in **Table S1**.
