# Supplementary material for: Distantly related Alteromonas bacteriophages share tail fibers exhibiting properties of transient chaperone caps
Source: Nat Commun. 2023 Oct 16;14:6517. doi: 10.1038/s41467-023-42114-8 (PMC10579305; doi:10.1038/s41467-023-42114-8)
Supplement: Supplementary file 3 — Reporting Summary [file 41467_2023_42114_MOESM3_ESM.pdf]

nature portfolio

Corresponding author(s): Matthew Dunne

Last updated by author(s): Sep 4, 2023

Reporting Summary

Nature Portfolio wishes to improve the reproducibility of the work that we publish. This form provides structure for consistency and transparency in reporting. For further information on Nature Portfolio policies, see our Editorial Policies and the Editorial Policy Checklist.

Statistics

For all statistical analyses, confirm that the following items are present in the figure legend, table legend, main text, or Methods section.

n/a	Confirmed	
x	☐	The exact sample size (*n*) for each experimental group/condition, given as a discrete number and unit of measurement
☐	x	A statement on whether measurements were taken from distinct samples or whether the same sample was measured repeatedly
x	☐	The statistical test(s) used AND whether they are one- or two-sided<br>*Only common tests should be described solely by name; describe more complex techniques in the Methods section.*
x	☐	A description of all covariates tested
x	☐	A description of any assumptions or corrections, such as tests of normality and adjustment for multiple comparisons
x	☐	A full description of the statistical parameters including central tendency (e.g. means) or other basic estimates (e.g. regression coefficient) AND variation (e.g. standard deviation) or associated estimates of uncertainty (e.g. confidence intervals)
x	☐	For null hypothesis testing, the test statistic (e.g. *F*, *t*, *r*) with confidence intervals, effect sizes, degrees of freedom and *P* value noted<br>*Give P values as exact values whenever suitable.*
x	☐	For Bayesian analysis, information on the choice of priors and Markov chain Monte Carlo settings
x	☐	For hierarchical and complex designs, identification of the appropriate level for tests and full reporting of outcomes
x	☐	Estimates of effect sizes (e.g. Cohen's *d*, Pearson's *r*), indicating how they were calculated

Our web collection on statistics for biologists contains articles on many of the points above.

Software and code

Policy information about availability of computer code

Data collection

A5 genome sequencing data was obtained with Illumina MiSeq (2 x 300 bp). Genome extraction and analysis is sufficiently described in the Methods section.

Data analysis

FastQC (version 0.11.7)
Trimmomatic (version 0.32 61)
SPAdes (version 3.12.0)
Bowtie2 (version 1.3.1)
SAMtools (version 1.2)
Tablet (https://ics.hutton.ac.uk/tablet/)
Prodigal (version 2.60)
Diamond (version 0.9.4.105)
hmmscan (HMMER server; version 3.1b2)
Easyfig (version 2.2.2)
Flex2 (in-house python software for genome comparative analysis)
MultAlin (http://multalin.toulouse.inra.fr/multalin/) version 5.4.1
MUSCLE (https://www.ebi.ac.uk/Tools/msa/muscle/) version n/A
IQ-TREE (version 1.6.11) from ModelFinder (http://www.iqtree.org/ModelFinder/)
iTOL (version 4)
BLAST (https://blast.ncbi.nlm.nih.gov/Blast.cgi) versions 2.12.0 and 2.13.0

Fiji ImageJ (version 2.0.0)
GraphPad Prism (Version 9)
AlphaFold 2.0 and AlphaFold-Multimer (https://github.com/deepmind/alphafold)
PyMol (Version 2.5.3)
HHpred (https://toolkit.tuebingen.mpg.de/tools/hhpred) Version 57c8707149031cc9f8edceba362c71a3762bdbf8
DALI protein structure comparison server (http://ekhidna2.biocenter.helsinki.fi/dali/) Version DaliLite.v5
Inkscape (Version 1.1)

For manuscripts utilizing custom algorithms or software that are central to the research but not yet described in published literature, software must be made available to editors and reviewers. We strongly encourage code deposition in a community repository (e.g. GitHub). See the Nature Portfolio guidelines for submitting code & software for further information.

Data

Policy information about availability of data

All manuscripts must include a data availability statement. This statement should provide the following information, where applicable:

- Accession codes, unique identifiers, or web links for publicly available datasets
- A description of any restrictions on data availability
- For clinical datasets or third party data, please ensure that the statement adheres to our policy

All Source Data has been provided with this manuscript. A data availability statement is included too: "Annotated genomes of vB_AmeP_A5 and its host A. mediterranea PT15 are available from the GenBank database under accession numbers OP481051 and NZ_CP041170, respectively. Alteromonas phage vB_AmeP_V19 accession number is OP751378. Source data are provided with this paper."

Hyperlinks are provided throughout the main text, supplementary information, and in the figure legends for all GenBank and PDB entries.

Public databases that were also accessed during sequence analysis and mentioned in the Methods section:
NCBI (http://www.ncbi.nlm.nih.gov/RefSeq/)
pVOGs (doi: 10.1093/nar/gkw975)
Pfam/InterPro (www.ebi.ac.uk/interpro)
CDD/SPARCLE (doi: 10.1093/nar/gkz991)

Research involving human participants, their data, or biological material

Policy information about studies with human participants or human data. See also policy information about sex, gender (identity/presentation), and sexual orientation and race, ethnicity and racism.

Reporting on sex and gender	This paper contains no data involving human participants, their data, or biological material
Reporting on race, ethnicity, or other socially relevant groupings	This paper contains no data involving human participants, their data, or biological material
Population characteristics	This paper contains no data involving human participants, their data, or biological material
Recruitment	This paper contains no data involving human participants, their data, or biological material
Ethics oversight	This paper contains no data involving human participants, their data, or biological material

Note that full information on the approval of the study protocol must also be provided in the manuscript.

Field-specific reporting

Please select the one below that is the best fit for your research. If you are not sure, read the appropriate sections before making your selection.

☒ Life sciences ☐ Behavioural & social sciences ☐ Ecological, evolutionary & environmental sciences

For a reference copy of the document with all sections, see nature.com/documents/nr-reporting-summary-flat.pdf

Life sciences study design

All studies must disclose on these points even when the disclosure is negative.

Sample size

No statistical method was required to predetermine sample sizes for our experiments as our study focuses on (i) characterizing a specific host recognition module shared by a limited number of Alteromonas phages that had publicly accessible genomes at the time, and (ii) the characterization of the four phage tail fibers and their respective chaperones that are studied in this paper.

Nevertheless, there is one incidence were sample size was chosen based on practical considerations and previous studies in the field as the Alteromonas isolates (n=10) used for host range testing of phage A5 in Table 1 were selected based on the host-range analysis performed with the myophage V22 in a previous study (https://doi.org/10.1128/mSystems.00217-20). Infectivity was observed

Data exclusions	No data was excluded.
Replication	A single transmission electron microscopy image of phages A5 and V22 (Fig. 2c) was selected from multiple micrographs taken from a single phage-coated grid as is common practice. Phage infectivity assessment was performed at least two times producing the same results. Fluorescence microscopy was independently repeated at least two times per experiment (protein + strain) providing similar results that were equivalent to previous observations (https://doi.org/10.1128/mSystems.00217-20). SEC chromatograms were collected once under specified conditions in a single session, with each protein run in consecutive order. Analogous results were observed independently and matched previous observations for V22 gp26-27 (https://doi.org/10.1128/mSystems.00217-20).
Randomization	There was no requirement for randomization. All samples and data points were analyzed and reported equally.
Blinding	Blinding was not necessary as all data collected and analyzed involved objective measurements with no subjective judgments or biases. We knew at all times which sequences were being aligned, which proteins were being predicted with AlphaFold, and which samples were being analyzed.

Reporting for specific materials, systems and methods

We require information from authors about some types of materials, experimental systems and methods used in many studies. Here, indicate whether each material, system or method listed is relevant to your study. If you are not sure if a list item applies to your research, read the appropriate section before selecting a response.

Materials & experimental systems

n/a	Involved in the study
☒	☐ Antibodies
☒	☐ Eukaryotic cell lines
☒	☐ Palaeontology and archaeology
☒	☐ Animals and other organisms
☒	☐ Clinical data
☒	☐ Dual use research of concern
☒	☐ Plants

Methods

n/a	Involved in the study
☒	☐ ChIP-seq
☒	☐ Flow cytometry
☒	☐ MRI-based neuroimaging
